# Supplementary material for: Pyruvate Dehydrogenase Kinase 1 inhibition mediated oxidative phosphorylation enhancement in cartilage promotes osteoarthritis progression
Source: BMC Musculoskelet Disord. 2023 Jul 20;24:597. doi: 10.1186/s12891-023-06585-6 (PMC10357736; doi:10.1186/s12891-023-06585-6)
Supplement: Supplementary file 1 — Additional file 1. [file 12891_2023_6585_MOESM1_ESM.zip › MF.Gsea.1653627316242/GOMF_ARACHIDONIC_ACID_MONOOXYGENASE_ACTIVITY.html]

Details for gene set GOMF\_ARACHIDONIC\_ACID\_MONOOXYGENASE\_ACTIVITY[GSEA]

|  || Dataset | 55457\_collapsed\_to\_symbols.cls.cls #OA\_versus\_normal.cls.cls #OA\_versus\_normal\_repos |
| Phenotype | cls.cls#OA\_versus\_normal\_repos |
| Upregulated in class | normal |
| GeneSet | GOMF\_ARACHIDONIC\_ACID\_MONOOXYGENASE\_ACTIVITY |
| Enrichment Score (ES) | -0.554228 |
| Normalized Enrichment Score (NES) | -1.5891201 |
| Nominal p-value | 0.026711185 |
| FDR q-value | 0.15655145 |
| FWER p-Value | 0.998 |
Table: GSEA Results Summary

  

Fig 1: Enrichment plot: GOMF\_ARACHIDONIC\_ACID\_MONOOXYGENASE\_ACTIVITY      
 Profile of the Running ES Score & Positions of GeneSet Members on the Rank Ordered List

  

| SYMBOL | TITLE | RANK IN GENE LIST | RANK METRIC SCORE | RUNNING ES | CORE ENRICHMENT || 1 | CYP4F11 | cytochrome P450 family 4 subfamily F member 11 [Source:HGNC Symbol;Acc:HGNC:13265] | 5144 | 0.060 | -0.3834 | No |
| 2 | CYP2C19 | cytochrome P450 family 2 subfamily C member 19 [Source:HGNC Symbol;Acc:HGNC:2621] | 6089 | -0.011 | -0.4536 | No |
| 3 | CYP2C18 | cytochrome P450 family 2 subfamily C member 18 [Source:HGNC Symbol;Acc:HGNC:2620] | 6210 | -0.022 | -0.4572 | No |
| 4 | CYP2B6 | cytochrome P450 family 2 subfamily B member 6 [Source:HGNC Symbol;Acc:HGNC:2615] | 7445 | -0.114 | -0.5230 | Yes |
| 5 | CYP2A6 | cytochrome P450 family 2 subfamily A member 6 [Source:HGNC Symbol;Acc:HGNC:2610] | 7849 | -0.144 | -0.5163 | Yes |
| 6 | CYP2F1 | cytochrome P450 family 2 subfamily F member 1 [Source:HGNC Symbol;Acc:HGNC:2632] | 7907 | -0.148 | -0.4818 | Yes |
| 7 | CYP1A1 | cytochrome P450 family 1 subfamily A member 1 [Source:HGNC Symbol;Acc:HGNC:2595] | 8195 | -0.171 | -0.4591 | Yes |
| 8 | CYP2C9 | cytochrome P450 family 2 subfamily C member 9 [Source:HGNC Symbol;Acc:HGNC:2623] | 8595 | -0.201 | -0.4372 | Yes |
| 9 | CYP4A11 | cytochrome P450 family 4 subfamily A member 11 [Source:HGNC Symbol;Acc:HGNC:2642] | 8654 | -0.205 | -0.3877 | Yes |
| 10 | CYP4F3 | cytochrome P450 family 4 subfamily F member 3 [Source:HGNC Symbol;Acc:HGNC:2646] | 9224 | -0.250 | -0.3660 | Yes |
| 11 | CYP4F2 | cytochrome P450 family 4 subfamily F member 2 [Source:HGNC Symbol;Acc:HGNC:2645] | 9599 | -0.283 | -0.3204 | Yes |
| 12 | CYP2J2 | cytochrome P450 family 2 subfamily J member 2 [Source:HGNC Symbol;Acc:HGNC:2634] | 9642 | -0.287 | -0.2481 | Yes |
| 13 | CYP2A13 | cytochrome P450 family 2 subfamily A member 13 [Source:HGNC Symbol;Acc:HGNC:2608] | 10732 | -0.397 | -0.2281 | Yes |
| 14 | CYP2E1 | cytochrome P450 family 2 subfamily E member 1 [Source:HGNC Symbol;Acc:HGNC:2631] | 10988 | -0.428 | -0.1351 | Yes |
| 15 | CYP4F12 | cytochrome P450 family 4 subfamily F member 12 [Source:HGNC Symbol;Acc:HGNC:18857] | 11423 | -0.490 | -0.0396 | Yes |
| 16 | CYP2C8 | cytochrome P450 family 2 subfamily C member 8 [Source:HGNC Symbol;Acc:HGNC:2622] | 11958 | -0.585 | 0.0731 | Yes |
Table: GSEA details [plain text format]

  

Fig 2: GOMF\_ARACHIDONIC\_ACID\_MONOOXYGENASE\_ACTIVITY      
 Blue-Pink O' Gram in the Space of the Analyzed GeneSet

  

Fig 3: GOMF\_ARACHIDONIC\_ACID\_MONOOXYGENASE\_ACTIVITY: Random ES distribution      
 Gene set null distribution of ES for **GOMF\_ARACHIDONIC\_ACID\_MONOOXYGENASE\_ACTIVITY**
